# Sources of free sugar in the diet of Saudi children

**DOI:** 10.3389/fpubh.2024.1305364

**Published:** 2024-04-09

**Authors:** Walaa Abdullah Mumena, Hebah Alawi Kutbi

**Affiliations:** ^1^Clinical Nutrition Department, College of Applied Medical Sciences, Taibah University, Madinah, Saudi Arabia; ^2^, Faculty of Applied Medical Sciences, King Abdulaziz University, Jeddah, Saudi Arabia

**Keywords:** sugars, child, sugar-sweetened beverages, public health, Saudi Arabia

## Abstract

**Introduction:**

Data concerning sources of free sugar (FS) in the diet of Saudi children are limited. Identification of the top sources of FS would permit to develop tailored interventions that aid in meeting the recommendations of the World Health Organization for FS (≤ 25 g/day). This study aimed to investigate sources of FS in children’s diet.

**Methods:**

A cross-sectional data of healthy children ages between 6 and 12 years who reside in Saudi Arabia were gathered from their mothers using online platforms (WhatsApp, Facebook, and X) (*n* = 424; 210 boys and 214 girls). An interview was conducted through the phone with the mother and her child to collect data pertaining FS intake using a validated food frequency questionnaire.

**Results:**

The primary sources of FS were Sweetened Beverages, followed by Sugars, Sweet Bakery Products, Other Desserts, and Candies, of which the added sugar consisted mostly of its total sugar. The consumption of FS coming from solid food sources for the highest quartile of children almost doubled the amount of FS coming from liquid food sources. Compared to boys, girls in the top quartile of FS intake consumed significantly higher amounts of solid sugars (*p* = 0.030). Only the intake of FS coming from Sweetened Beverages was associated negatively with age of children (*p* = 0.032).

**Discussion:**

Public health interventions should emphasize the necessity of reducing the consumption of the top sources of FS to practically achieve the recommendations of FS intake.

## Introduction

In the recent years, added sugar intake has been of interest among researchers and public health organizations (1, 2). High consumption of added sugars has been frequently reported among children and adults in several settings (3–6), particularly in the form of sugar-sweetened beverages (7–9). The term added sugar refers to “sugars and syrups added to foods during processing or manufacturing, and sugars added at the table” (10). Excessive added sugar consumption was found to be associated with excess body weight and increased risk of a number of non-communicable diseases, including obesity, type-2 diabetes, and cardiovascular diseases (11). This had led many public health agencies, such as the Centers for Disease Control and Prevention and American Heart Association, to develop dietary recommendations aiming to limit added sugar consumption (10, 12).

Supported by the latest evidence, the World Health Organization (WHO) announced a new guideline in 2015 targeting free sugars (FSs), recommending children and adults to limit the daily consumption of FS to <10% of total energy, with a further reduction to <5% or 25 g per day for additional health benefits (13, 14). FSs refer to mono- and disaccharides added to foods during manufacturing, cooking, or consuming, in addition to sugars that naturally exist in fruit juices, syrups, and honey (14). Growing numbers of health organizations are focusing on FS including the Scientific Advisory Committee on Nutrition, UK (15) and the European Food Safety Authority (16).

Despite the negative impact of FSs on health, some argues that food sources may matter more than the total intake, particularly for children (17). For instance, many children’s favorite foods, such as discretionary foods (i.e., biscuits and cakes), can be poor in nutrients and contain large amounts of FSs; However, other foods containing FSs could be considered good sources of key nutrients, such as flavored milk (18) and fortified breakfast cereals (19). It has been further suggested that adding sugar to some unpalatable but nutritious foods could promote consumption of these foods among children (20). In fact, different interventions have been implemented in different settings targeting excessively consumed sources of FSs (21).

Several recent programs/polices have been established to reduce free/added sugar intake among the Saudi population including excise tax on sugary drinks, added sugar content in nutrition fact labels, and sugar awareness campaign (22–24); however, national data concerning sources of FS are still lacking. In order to plan tailored interventions, food sources that contribute greatly to the intake of FS in Saudi children must be recognized and targeted for reduction. Thus, we aimed in this study to identify top sources of FS in the diet of children in Saudi Arabia.

## Materials and methods

### Data collection

We recruited 539 mothers of Saudi children ages between 6 and 12 years between October 2021 and January 2022 Table 1. Data of children residing outside of Saudi Arabia, with food allergies, or chronic diseases were excluded. Data were collected conveniently using river sampling technique. A survey invitation link accompanied by a text message describing study objectives, the inclusion criteria, and consent for participation was distributed in several social media platforms that are commonly used in the Saudi community, including WhatsApp, Facebook, and X. Additional statement requesting circulation of the invitation link was also included. The minimum number of participants needed for this study was 323 mothers based on an expected proportion of children who consume FS excessively of 30%, precision of 5, and 95% confidence level (25). Consents for participation and sociodemographic information were provided by the mothers via the online survey. Contact information of mothers was also collected to schedule a phone interview to collect dietary data of children.

**Table 1:** Characteristics of children and their mothers stratified by children’s sex (*n* = 424).

Variable	Boys (*n* = 210)	Girls (*n =* 214)	*p*-value
**Age group**			**0.208**
6–7 years	79 (54.9)	65 (45.1)	
8–9 years	56 (44.4)	70 (55.6)	
10–12 years	75 (48.7)	79 (51.3)	
**Order of child between siblings**			
Only child	11 (50.0)	11 (50.0)	**0.703**
Older child	68 (48.9)	71 (51.1)	
Middle child	72 (53.3)	63 (46.7)	
Youngest child	59 (46.1)	69 (53.9)	
**Maternal age group**			
≤ 30 years	35 (46.1)	41 (53.9)	**0.765**
31–40 years	119 (49.8)	120 (50.2)	
> 40 years	56 (51.4)	53 (48.6)	
**Maternal education level**			**0.253**
≤ High school	45 (42.9)	60 (57.1)	
Bachelor’s degree	138 (51.1)	132 (48.9)	
Postgraduate degree	27 (55.1)	22 (44.9)	
**Family monthly income, in Saudi Riyals**			
< 4,000	16 (55.2)	13 (44.8)	**0.791**
4,000–10,000	80 (48.5)	85 (51.5)	
> 10,000	114 (49.6)	116 (50.4)	

Alpha = 0.05. Data presented in table obtained using Fisher’s exact test.

This study was conducted according to the guidelines laid down in the Declaration of Helsinki and all procedures involving human subjects were approved by the Ethical Review Board at the College of Applied Medical Sciences, Taibah University [certificate# 2020/55/202/CLN]. Written informed consent was obtained from all mothers.

### Sample characteristics

Data concerning children’s age (in years) based on date of birth, sex (boys; girls), order of child among siblings (only child; older child; middle child; younger child), maternal age in years, maternal education level (≤high school; bachelor’s degree; postgraduate degree), and family monthly income in Saudi Riyals (less than 4,000; 4,000–10,000; more than 10,000) were collected via the online survey.

### Assessment of food sources of free sugar

Mothers were contacted by trained data collection personnel to schedule for a phone interview with the mother in order to complete the food frequency questionnaire (FFQ). The child and any individual who supervise the child during eating was requested to attend the interview. A food guide as well as a table of frequency response options were shared with mothers via WhatsApp to help in accurately estimating the portion size of foods consumed. A validated FFQ that has been designed to evaluate FS intake among Saudi children was used to investigate the top sources of FS (26). The FFQ includes 40 food items classified in 12 food groups as follows: (1) “sweetened beverages (fruit drink, soft drink, flavored milk, smoothie)”; (2) “ready to eat cereals (ready to eat cereals: lower sugar [≤ 21.2 g/100 g], ready to eat cereals: higher sugar [> 21.2 g/100 g])”; (3) “breads and rolls (yeast breads, rolls and buns)”; (4) “sweet bakery products (cake, fruit pie or cheesecake, cookies, brownies, doughnuts, biscuit, muffins, French toast, pancakes/waffles, sweet pastries)”; (5) “quick breads and bread products (croissant, pastries)”; (6) “candy (candy not containing chocolate; candy containing chocolate)”; (7) “other desserts (ice-cream, popsicle, gelatin, pudding)”; (8) “sugars (sugars in tea and coffee, honey, jam, chocolate spread, peanut butter, syrups)”; (9) “yogurt (yogurt, flavored yogurt)”; (10) “mixed dishes (pizza or burger, all varieties, Chinese food, all varieties)”; (11) “condiments and sauces (ketchup, salad dressing [ranch, blue cheese, and Italian], salad dressing: [French, BBQ, and thousand island])”; (12) “fruits (canned fruits).” Frequency of consumption were as follows: “per day (once, 2–3 times, 4–5 times, or 6 times or more)”, “per week (once, 2–4 times, or 5–6 times)”, “per month (< once or 1–3 times)”. Responses were recorded into a hard copy of the FFQ then entered into excel sheet to auto-calculate quantities of FS consumed in grams per day from each food item.

### Statistical analysis

Data concerning FS intake are presented as mean ± standard deviation (SD) or frequency and percentage. FS intake was compared to the WHO recommendation of ≤25 g/day (13). Top sources of FSs were identified based on the mean intake of each food group in the highest quartile of FS intake. Fisher’s exact test was used to compare the proportions across the different groups (boys vs. girls). Mann–Whitney U test was performed to compare the average intake of FS among boys and girls. Multiple linear regression analysis was used to explore the association between FS intake (dependent variable) and age of children (independent variable) in the different food sources of FS controlling for children’s sex. A significant level of 95% was used to assess significance of tests performed. Data in this study were analyzed using the Statistical Package for the Social Sciences (SPSS) version 20 (SPSS, Inc., Chicago, IL, USA).

## Results

The number of participants included in this study is 424, after excluding children food allergy, chronic disease, and incomplete dietary data (*n* = 115, 21.3%). Nearly half of the children included in the study were girls (*n* = 214, 50.5%). Mean age of children was 8.67 ± 1.85 years, while same proportion of boys and girls reported as the only child in the household (*n* = 11, 5.19%, each sex). Most of the mothers were over 30 years of age (*n* = 348, 82.1%), whereas most of the mothers reported holding a bachelor’s degree or higher (*n* = 319, 75.2%). Over half of the sample report a family income of > SR 10,000 per month (*n* = 229). Characteristics of boys and girls included in this study were similar (*p* > 0.05). Detailed description of the characteristics of the study sample is illustrated in Table 1.

The mean intake of FS exceeds the WHO recommendation of ≤25 g/day of FS (94.5 ± 52.9 g/day). Mean intake of FS of children stratified by sex and quartile of intake is illustrated in Table 2. Boys in the 1st quartile exceed the recommendation by 15.7 g/day (62.8%), whereas girls in the 1st quartile exceed the recommendation by 16.5 g/day (66.0%). Boys in the 4th quartile exceed the recommendation by 132 g/day (528%), whereas girls in the 4th quartile exceed the recommendation by 136 g/day (544%). Average intake of FS was similar among boys and girls in all quartile groups (*p* > 0.05).

**Table 2:** Mean intake of free sugar among children, stratified by sex and quartile of intake.

Quartile	Boys (*n* = 210)		Girls (*n* = 214)			Total (*n* = 242)	
	FS intake g/day	FS intake kcal/day	FS intake g/day	FS intake kcal/day	*p*-value	FS intake g/day	FS intake kcal/day
Q1 (*n* = 91)	40.7 ± 9.15	163 ± 36.6	41.5 ± 10.8	166 ± 43.2	0.449	41.2 ± 10.1	165 ± 40.4
Q2 (*n* = 107)	67.6 ± 7.42	270 ± 29.7	67.6 ± 7.39	270 ± 29.6	0.995	67.6 ± 7.37	270 ± 29.5
Q3 (*n* = 112)	95.8 ± 8.85	383 ± 35.4	95.5 ± 8.84	382 ± 35.4	0.900	95.6 ± 8.81	382 ± 35.2
Q4 (*n* = 112)	157 ± 46.7	628 ± 187	161 ± 45.8	644 ± 183	0.433	159 ± 46.1	636 ± 184

Alpha = 0.05. Data presented in table obtained using Mann–Whitney U test. FS: free sugar.

**Figure 1:**
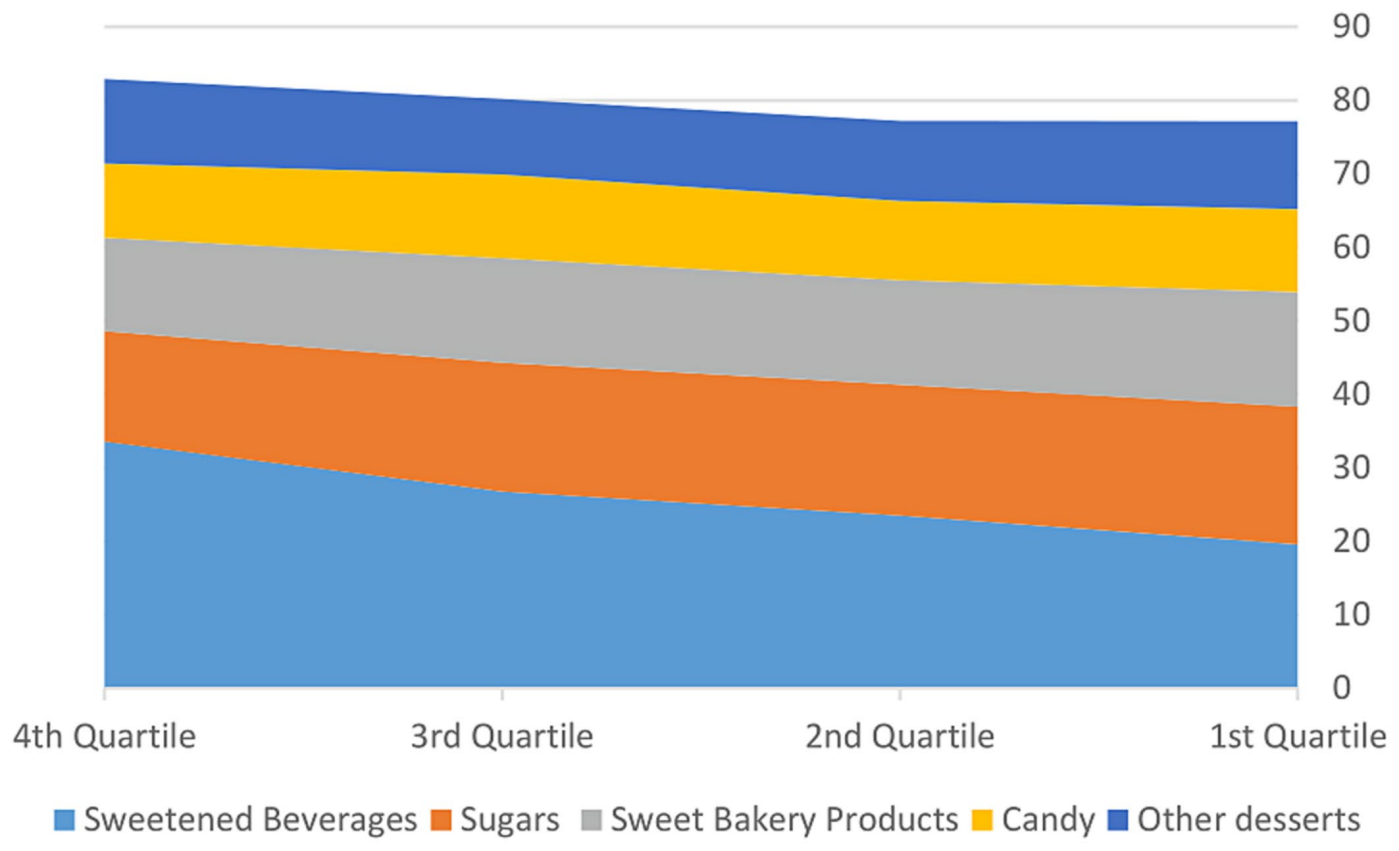
Top sources of free sugar consumed by Saudi children ages 6–12 years (g/day).

Top sources of FS consumed (in grams) by the children included in this study are presented in Figure 1. The top two sources of FS consumed include Sweetened Beverages (soft drink; fruit drink; flavored milk; smoothie) with a mean intake of 27.2 g/day, followed by Sugars (honey; sugars in tea and coffee; chocolate spread; jam; syrups; peanut butter;), with a mean intake of 15.4 g/day. The next three top food sources of FS are Sweet Bakery Products (cake; fruit pie or cheesecake; brownies; cookies; doughnuts; biscuit; muffins; French toast; pancakes/waffles; sweet pastries), with a mean intake of 12.8 g/day, Other Desserts (popsicle; ice-cream; gelatin; pudding, with a mean intake of 10.7 g/day, and Candy (candy not containing chocolate; candy containing chocolate), with a mean intake of 10.0 g/day). In the top quartile of FS intake, girls consumed significantly higher quantitates of FS coming from honey; sugars in tea and coffee; chocolate spread; jam; syrups; peanut butter compared to boys (26.8 ± 17.9 g/day vs. 21.5 ± 16.7 g/day, respectively, *p* = 0.021). Data concerning intake of FSs for children in 1st and 4th quartile of FS intake are presented in Table 3.

**Table 3:** Top five food sources of free sugars for Saudi children in 1st and 4th quartile of free sugar intake (*n* = 424).

Rank	1st Quartile				4th Quartile			
	Boys		Girls		Boys		Girls	
	Food group	FS from food group, g	Food Group	FS from food group, g	Food group	FS from food group, g	Food group	FS from food group, g
1	Sweetened beverages	8.89 ± 6.14	Sweetened beverages	8.47 ± 7.22	Sweetened beverages	63.1 ± 37.4	Sweetened beverages	49.2 ± 36.5
2	Sugars	7.87 ± 5.74	Sugars	7.39 ± 6.79	Sugars	21.5 ± 16.7	Sugars*	26.8 ± 17.9
3	Sweet bakery products	6.26 ± 4.66	Sweet bakery products	6.58 ± 4.06	Sweet bakery products	20.6 ± 11.2	Other desserts	21.5 ± 17.5
4	Candy	4.76 ± 4.30	Other desserts	5.23 ± 4.42	Other desserts	18.4 ± 18.1	Sweet bakery products	20.7 ± 17.7
5	Other desserts	4.69 ± 4.94	Yogurt	5.07 ± 3.82	Candy	16.2 ± 16.8	Candy	17.1 ± 13.7

Numbers presented in table are mean ± SD. * Alpha = 0.05. FS: free sugar.

**Figure 2:**
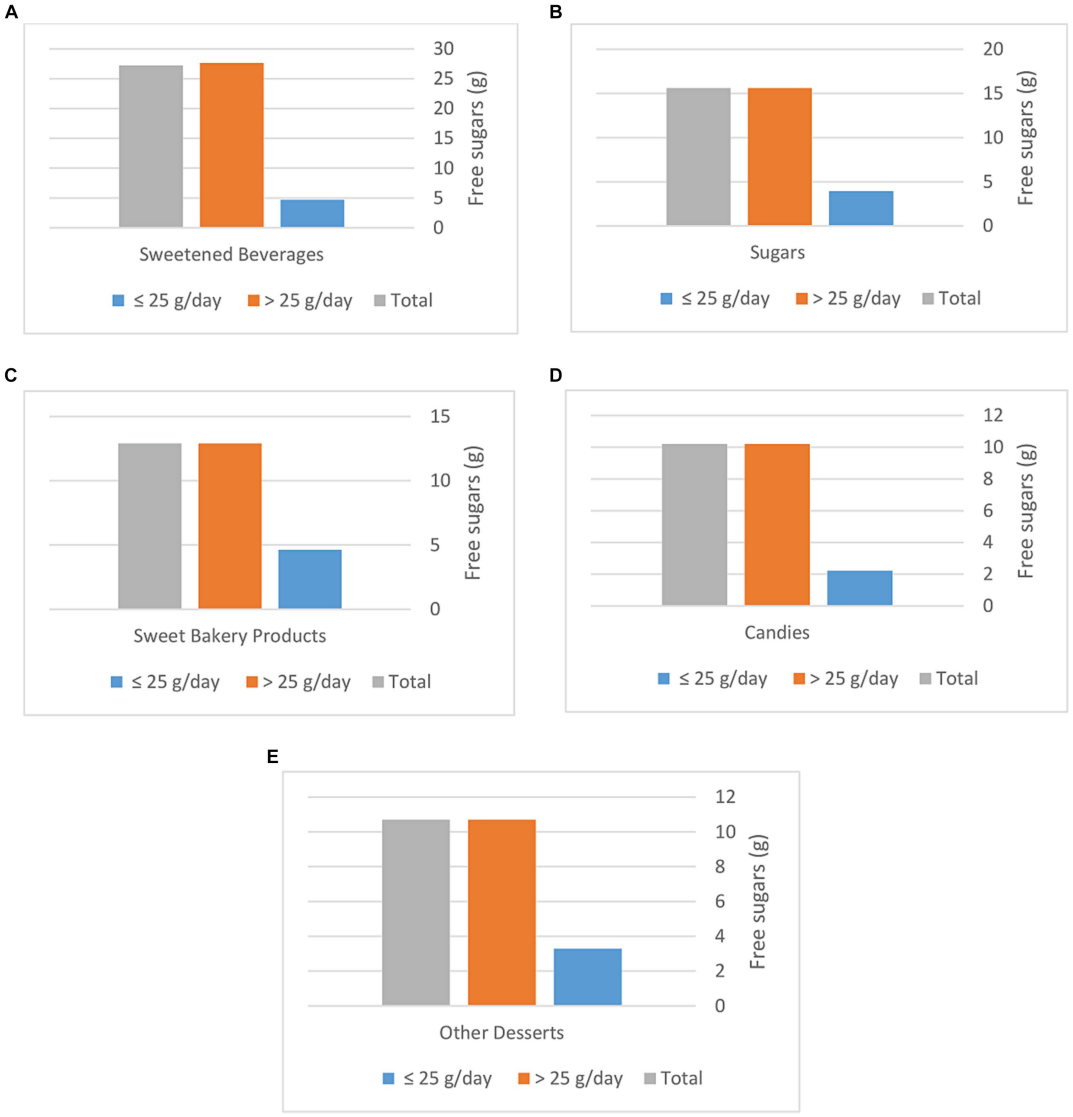
Mean intake of free sugar (g/day) for the top sources of free sugar consumed by Saudi children ages 6–12 years (Sweetened Beverages **(A)**; Sugars **(B)**; Sweet Bakery Products **(C)**; Candies **(D)**; Other Desserts **(E)**) based on the WHO recommendation (≤ 25 g/day vs. > 25 g/day).

Figure 2 presents the mean intake of FS (g/day) for the top sources of FS consumed by Saudi children (Sweetened Beverages (a); Sugars (b); Sweet Bakery Products (c); Candies (d); Other Desserts (e)) based on the WHO recommendation (≤ 25 g/day vs. > 25 g/day). Mean intake of FS from Sweetened Beverages for children who exceeded the WHO recommendation was 27.6 ± 28.0 g/day; Sugars 15.6 ± 13.5 g/day; Sweet Bakery Products 12.9 ± 10.8 g/day; Candies 10.2 ± 10.6 g/day; Other Desserts 10.7 ± 12.2 g/day.

Multiple linear regression analysis conducted to explore the association between FS intake and children’s age adjusting for child’s sex indicated no association between the consumption of FS and age of children (age in years) (Beta (B) = −2.28, Standard Error (SE) = 1.39, 95% Confidence Interval (CI): [−5.01 to 0.45], *p* = 0.102). However, only the intake of FS coming\from Sweetened Beverages was negatively linked to age of children (B = −1.57, SE = 0.73, 95% CI: [−3.01 to −0.14], *p* = 0.032). Age was not linked to consumption of FS from other foods (sugars; sweet bakery products; candy) (Table 4).

**Table 4:** Multiple linear regression analysis of the association between free sugar intake in g/day and age of children.

	Beta	Standard error	*p*-value	95% Confidence interval	R-square
Sweetened beverages	−1.57	0.73	0.032*	−3.01 to −0.14	0.02
Sugars	0.51	0.36	0.151	−0.19 to 1.21	0.01
Sweet bakery products	−0.55	0.28	0.054	−1.11 to 0.01	0.01
Candy	−0.37	0.28	0.181	−0.92 to 0.18	0.01
Other desserts	−0.62	0.32	0.052	−1.24 to 0.01	0.01

*Alpha = 0.05. Models were all adjusted for children’s sex.

Mean FS intake from liquid sources for the highest quartile is 54.6 ± 32.6 g/day, while mean FS for the highest quartile from solid food sources is 105 ± 41.4 g/day. Thirty-four percent of FS intake for the highest quartile is coming from liquid sources. In the top quartile of FS intake, girls consumed significantly higher quantities of FS coming from solid food sources compared to boys (112 ± 41.4 g/day vs. 99.2 ± 40.7 g/day, respectively, *p* = 0.030) (Table 5).

**Table 5:** Free sugar intake (liquid sources and solid sources) of boys and girls by quartile of free sugar intake (*n* = 424).

FS intake	1st Quartile				4th Quartile			
	Boys	Girls	*p*-value	Total	Boys	Girls	*p*-value	Total
FS intake from liquid sources, g/day	7.20 ± 5.10	8.28 ± 6.63	0.723	7.83 ± 6.03	58.7 ± 28.5	50.1 ± 36.5	0.049	54.6 ± 32.6
FS intake from solid food sources, g/day	33.5 ± 8.51	33.4 ± 9.49	0.885	33.4 ± 9.05	99.2 ± 40.7	112 ± 41.4	0.030*	105 ± 41.4
Total FS intake, g/day	40.7 ± 9.15	41.5 ± 10.8	0.449	41.2 ± 10.1	157 ± 46.7	161 ± 45.8	0.433	159 ± 46.1

* Alpha = 0.05. Data presented in table obtained using Mann–Whitney U test. FS: free sugar.

## Discussion

Our data show excessive consumption of FS among the children; Even the lowest quartile of children’s intake exceed the WHO recommendation of ≤ 25 g/day. The primary sources of FS consumed by the children include Sweetened Beverages, followed by Sugars, Sweet Bakery Products, Desserts (e.g., ice-cream, popsicle, gelatin, pudding), and Candies. The consumption of FS coming from solid foods in the highest quartile of children almost doubles the amount of FS from liquid food sources. Girls in the top quartile of FS intake consumed significantly higher amounts of solid sugars. Intake of FS coming only from Sweetened Beverages was associated negatively with children’s age.

Previous work has mainly focused on identifying top food sources of added sugars (5, 6). One cohort study conducted in eight European countries reported that fruit juices and soft drinks were the top contributors to FS intake among children ages between 2 and 9 years (27). Shifted attention to the consumption of FS has been noticed after the recent WHO recommendation. Added sugar are added to foods and drinks by the consumer, cook, or manufacturer; FSs, on the other hand, expand the definition of added sugar by including sugars naturally present in fruit juices, syrups, and honey (14). The recent recommendations were supported by the latest scientific evidence linking the intake of FS with excess body weight, non-communicable diseases, and dental caries (13, 15, 16). Therefore, implementation of public health interventions to limit FS consumption is vital. In fact, the population in Saudi Arabia were globally ranked the fifth largest consumers of sugar-sweetened beverages in 2015 (29). In 2018, the Saudi Food and Drug Authority required the inclusion of added sugars on nutrition fact labels in order to assist individuals in making informed choices based on their needs and preferences (23). In 2017, excise taxes have been implemented by the country on soft drinks and energy drinks, which increased the prices by 50 and 100%, respectively (30). Although implementation of this excise tax was followed by reduction of soft drink and energy drink sales (22), our data indicate that the mean intake of FS continues to be well above the recommendations. Modifying tax structure by including top sources of FS could maximize health benefit in Saudi Arabia. Furthermore, public health messages should emphasize that sugar is the targeted nutrient to reduce in order to limit potential substitution to other sugary foods or beverages.

Top sources of sugars vary across cultures and communities. For instance, data obtained from the National Health and Nutrition Examination survey (2009–2012) show that sweetened beverages, sweet bakery products, candies, and other desserts (i.e., pudding and frozen dairy desserts, ready to eat cereals, and flavored milk) are the primary sources of added sugars in the diet of American children (5). For Australian children, data obtained from the Australian National Children Nutrition and Physical Activity Survey (2007) indicated that sugar-sweetened beverages, other desserts category (i.e., pudding and frozen dairy desserts), chocolate and confectionary, sweetened dairy products, and desserts (i.e., cakes, biscuits, pastries) are the top sources of sugars, with a considerably lower intake reported of FS compared to that of Saudi children (83.5 ± 41.1 vs. 94.5 ± 52.9 g/day) (6). Of note, the sweetened beverages, or sugar-sweetened beverages, have been repeatedly shown to be highly consumed by children (5, 6, 32). Given that sweetened beverages are typically high in added sugar with poor nutrient density, their consumption have been associated with type-2 diabetes mellitus (31, 33) cardiovascular diseases (34, 35), and excess body weight (36).

Nevertheless, our data show that the intake of solid FSs among the children is nearly 2.5 times greater than that of liquid FSs. A few numbers of studies examined the influence of liquid vs. solid sugars on the risk of developing features of metabolic syndrome among children. For instance, a 2-year longitudinal study conducted on Canadian children showed that high intake of liquid added sugars predicted the risk of insulin resistance, whereas solid sugars (i.e., desserts and candies) did not; and neither of the sugar forms predicted changes in the weight status or waist circumference of the children (37). In a study of American children, the higher intake of liquid sugars predicted increased waist circumference, whereas increasing the intake of solid sugars was positively associated with the waist circumference only among children with obesity (38). Furthermore, a narrative review of studies evaluating the influence of liquid vs. solid sugars on the risk of metabolic syndrome suggested that liquid sources of sugars carry greater risk in terms of inducing features of metabolic syndrome compared to the solid forms (38). However, with the high consumption of FS observed among the children included in this study, there is certainly a scope to enhance their diet in terms of limiting the consumption of empty caloric foods and beverages; A practical aspect is to reduce the intake of the top sources of FS to help achieve the WHO recommendation of ≤ 25 g/day.

Strengths of the present study include being the first to report the top sources of FS among Saudi children and the use of an FFQ that has been validated to estimate the habitual intake of FS among the Saudi children. However, limitations of the study include the inability to collect data concerning energy intake using the FFQ, which are typically used to determine the contribution of FS to total energy intake. Therefore, data presented are based on the WHO recommendations per gram (≤ 25 g/day) solely. Data on dietary intake were collected using phone interviews, which may result in some bias related to reporting foods and portion estimates. Additionally, the convenient sampling may influence the generalizability of the study results.

In conclusion, public health interventions in Saudi Arabia should emphasize the necessity of reducing the consumption of Sweetened Beverages, followed by Sugars, Sweet Bakery Products, other Desserts (ice-cream; popsicle; gelatin; pudding), and Candies. Tailored messages to Saudi children, especially girls and young children, and their parents to limit the intake of FS and enhance diet quality are warranted. For instance, children may substitute water or milk for sweetened beverages; other food sources of FSs may be substituted with nutrient-dense healthy food options or used in smaller portions. Given that most of sugary foods and beverages are nutrient-poor, interventions which target limiting the intake of the top sources would reduce FS intake with minimal impact of core nutrients. Additionally, modifying tax structure by including the top sources of FS may maximize the health benefit for the population in Saudi Arabia. Future research should be directed toward investigating the top sources of FS among other age groups in Saudi Arabia.

## Data availability statement

The raw data supporting the conclusions of this article will be made available by the authors, without undue reservation.

## Ethics statement

The studies involving humans were approved by Ethical Review Board at the College of Applied Medical Sciences, Taibah University [certificate: 2020/55/202/CLN]. The studies were conducted in accordance with the local legislation and institutional requirements. Written informed consent for participation in this study was provided by the participants’ legal guardians/next of kin.

## Author contributions

WM: Conceptualization, Data curation, Formal analysis, Funding acquisition, Investigation, Methodology, Project administration, Resources, Software, Supervision, Validation, Visualization, Writing – original draft, Writing – review & editing. HK: Conceptualization, Data curation, Investigation, Methodology, Project administration, Resources, Software, Supervision, Validation, Visualization, Writing – original draft, Writing – review & editing.
